# Bax inhibitor-1 confers resistance to *Phytophthora parasitica* and is antagonized by RTP1 in Arabidopsis

**DOI:** 10.1007/s44154-026-00318-0

**Published:** 2026-06-23

**Authors:** Yujing Fang, Jing Zhang, Xianxian Gao, Shuhan Guo, Xiaoyin Xu, Bianbian Wang, Qing Zheng, Weixing Shan, Xiaoyu Qiang

**Affiliations:** State Key Laboratory for Crop Stress Resistance and High-Efficiency Production and College of Agronomy, Northwest A&F University, Yangling, Shaanxi 712100 China

**Keywords:** Plant immunity, Cell death, ER stress, *Phytophthora parasitica*, *RTP1*, *BI-1*

## Abstract

Plant immunity is closely intertwined with endoplasmic reticulum (ER) stress and regulated cell death. Here, we identify the ER-localized cell death regulator Bax Inhibitor-1 (BI-1) as a critical executor of resistance against *Phytophthora parasitica* in Arabidopsis, whose activity is antagonized by the susceptibility factor RTP1. RTP1 negatively modulates resistance to *P. parasitica* via ER stress and cell death regulation, yet its downstream coordinators remain unclear. We show that *BI-1* expression is significantly elevated in *rtp1* mutants during early colonization. Cell viability assays reveal *BI-1* is essential for *RTP1*-mediated cell death suppression, as *rtp1 bi-1* double mutants exhibit restored cell death. Pathogenicity assays demonstrate *BI-1* confers resistance required for the *rtp1*-mediated immunity: *rtp1 bi-1* mutants show increased susceptibility, reduced expression of ER stress-responsive immune genes (*WRKY33*, *CBP60g*, *MYB51*) and *PR1*, and abolished flg22-triggered ROS burst. Mechanistically, RTP1 interacts with BI-1 and reduces its protein accumulation through a proteasome-independent pathway, thereby antagonizing BI-1 function. Conversely, activated forms of ER stress transducers bZIP60 and bZIP28 bind and co-activate the *BI-1* promoter. Collectively, BI-1 serves as a key node in ER stress-associated immunity, post-translationally antagonized by RTP1 and transcriptionally driven by bZIP60/bZIP28. This study establishes the RTP1-BI-1 module as a pivotal interface linking ER stress perception to immune execution, offering a refined framework for engineering broad-spectrum disease resistance in crops.

**Supplementary Information:**

The online version contains supplementary material available at 10.1007/s44154-026-00318-0.

## Introduction

To detect potential invaders and respond appropriately, plants have evolved a complex and fine-tuned immune system that are often accompanied by a form of regulated cell death, such as hypersensitive response (HR) (Cook et al. 2015; Pitsili et al. 2020). Accumulating evidence demonstrates that plant immunity is intimately linked to cell death, and proteins that regulate cell death frequently play integral roles in plant immune response (Pitsili et al. 2020). The endoplasmic reticulum (ER), a key organelle housing cell death factors, is also a production site of antimicrobial proteins and immune signaling components, underscoring its importance in initiating programmed cell death (PCD) and modulating plant immunity (Eichmann and Schäfer 2012). Notably, the proteins involved in plant immunity with cell death (PICD), such as rice GF14e (Liu et al. 2016) and SPL33 (Wang et al. 2017), significantly contribute to plant resistance against a broad spectrum of pathogens, thus representing promising molecular targets for crop disease resistance breeding.

When confronted with stress stimuli, the demand for proper folding of secretory proteins in the ER can exceed its ER biosynthetic capacity, resulting in ER stress (Walter and Ron 2011; Park and Park 2019). To mitigate deleterious effects, a conserved cytoprotective signaling pathway known as unfolded protein response (UPR) is activated to restore ER homeostasis. In plants, the UPR is transduced through a well-characterized bipartite signaling module comprising the ER membrane-associated transducers inositol-requiring enzyme 1 (IRE1) and basic leucine zipper (bZIP) transmembrane transcription factors (e.g., bZIP60 and bZIP28 in Arabidopsis) (Howell 2013). However, severe or prolonged ER stress can redirect the UPR toward activating a cell death-triggering pathway. Accumulating evidence indicates that ER stress-induced cell death (ER-CD) signaling contributes significantly to stress adaptation (Ye et al. 2011; Jing et al. 2016; Guo et al. 2018; Gao et al. 2023), positioning modulators of ER-CD as attractive targets for engineering stress tolerance in plants. Nevertheless, the mechanisms coordinating pro-survival UPR and cell death signaling remain incompletely understood.

The ER-localized transmembrane protein Bax inhibitor-1 (BI-1) is a conserved cell death regulator in plants (Sanchez et al. 2000). Its involvement in plant defense responses, particularly in HR, has been well documented. Taking plant-fungal pathogen interactions as examples, overexpression of barley *BI-1* suppresses the cell death of host plants and promotes successful colonization of the biotrophic fungal pathogen *Blumeria graminis*, rendering barley more susceptible (Eichmann et al. 2010). Conversely, when confronted with necrotrophic fungal pathogen *Fusarium graminearum*, overexpression of *BI-1* confers enhanced resistance in barley (Babaeizad et al. 2008). The role of BI-1 in HR-mediated disease resistance against bacterial pathogens has also been demonstrated using Arabidopsis*-Pseudomonas syringae* pathosystem (Hogenkamp et al. 2010), and recent studies indicate that transient overexpression of potato *BI-1* in *Nicotiana benthamiana* enhances resistance against oomycete pathogen *Phytophthora* (Gao et al. 2022). Rather than acting in isolation, BI-1- mediated cell death regulation involves interactions with key modulators of Ca^2+^ signaling and lipid metabolism (Ishikawa et al. 2011; Nagano et al. 2019). Despite its pro-survival function, BI-1 can also interact with ATG6 to induce autophagy and PCD (Xu et al. 2017). Intriguingly, Arabidopsis BI-1 antagonizes the pro-survival function of bZIP28 during ER stress recovery, thereby modulating ER-CD signaling (Ruberti et al. 2018). This supports the notion that BI-1 acts in parallel with the UPR pathway to regulate ER stress-mediated cell death in Arabidospsis (Watanabe and Lam 2008).

Oomycete pathogen *Phytophthora* could infect various crops, including potato, soybean and tomato, causing devastating diseases. Taking potato late blight caused by *P. infestans* as an example, the rapid evolution of pathogen virulence often renders genotype-specific resistance genes ineffective, severely limiting sustainable potato production and leading to substantial economic losses (Fry 2008; Duan et al. 2023; Thines 2014). An effective strategy to confer durable disease resistance in crops is the modification of susceptibility genes (S genes), which are essential for pathogen compatibility (Pavan et al. 2010). Studies using the Arabidopsis-*P. parasitica* pathosystem have demonstrated that *P. parasitica* colonizes Arabidopsis roots more rapidly than leaves, with pathogen penetration occurring at 3 h post-inoculation (hpi), appressorium formation at 6 hpi, and haustorium-like structure development at 12 hpi, representing the biotrophic infection stages (Wang et al. 2011; Meng et al. 2015). Using this pathosystem, a number of Arabidopsis* S* genes have been identified (Li et al. 2019; Li et al. 2022; Lu et al. 2020; Pan et al. 2016; Yang et al. 2020, 2022). Among these, *RESISTANCE TO PHYTOPHTHORA PARASITICA 1* (*RTP1*), which encodes an ER membrane-localized protein, was characterized as a negative regulator of Arabidopsis resistance to multiple biotrophic pathogens. RTP1 modulates plant cell death, ROS production, and *PR1* expression during early infection stages (Pan et al. 2016). Moreover, *RTP1* participates in ER stress sensing by negatively regulating IRE1/bZIP60 splicing activity and stabilizes bZIP28. Upon *P. parasitica* infection, *rtp1bzip60* and *rtp1bzip28* mutant plants exhibit reduced resistance compared with *rtp1* plants, accompanied by attenuated induction of ER stress-responsive immune genes, suggesting that *rtp1*-mediated resistance is coordinately regulated with the UPR (Qiang et al. 2021).

We previously found that BI-1 physically interacts with RTP1 (Qiang et al. 2021). This prompted us to investigate the extent to which BI-1 contributes to RTP1-mediated cell death and immunity, and how the expression of *BI-1* is regulated by the bZIP60 and bZIP28 transcription factors. In this study, we demonstrate that *BI-1* not only plays an important role in the *RTP1*-mediated cell death, but is also vital for *rtp1*-mediated resistance against *P. parasitica* through modulating the expression of ER stress-responsive immune genes and ROS production. Notably, BI-1 protein stability is compromised through its interaction with RTP1. Furthermore, we show that the *BI-1* promoter is co-activated by and interacts with activated forms of bZIP28 and bZIP60 transcription factors, both of which potentiate *BI-1* transcription. These findings suggest divergent regulatory outputs of bZIP60 and bZIP28 in UPR and ER-CD signaling. Taken together, our work expands the mechanistic understanding of BI-1 in regulating ER stress-associated cell death and immunity through sophisticated coordination with the susceptibility factor RTP1. These insights not only advance our understanding of cell death in plant immunity, but also identify a promising pathway and molecular targets for crop disease resistance breeding.

## Results

### *BI-1* is significantly induced and affects cell death in *RTP1* loss-of-function mutant upon early colonization by *P. parasitica*

**Fig. 1:**
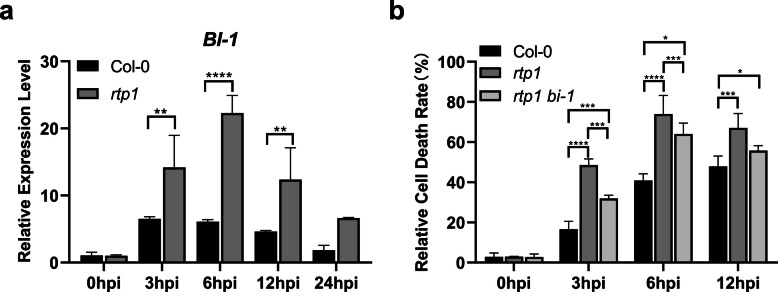
*BI-1* is significantly induced in *P. parasitica*-infected *rtp1* and affects *RTP1*-mediated cell death. **a** Expression levels of *BI-1* was determined by qRT–PCR. Roots of 2-wk-old plants of WT Col-0 and *rtp1* mutants were inoculated with *P. parasitica* zoospores or treated with mock solution. Total RNA was extracted from roots at 0, 3, 6, 12 and 24 hpi. *AtUBC9* was used as the plant reference gene. Three independent experiments were performed. Error bars indicate SD of three biological replicates. Asterisks indicate significance analyzed by Student’s t test (**P < 0.01; ****P < 0.0001). **b** At 0 (baseline, representing the mock/uninfected state), 3, 6 h, and 12 hpi post root inoculation with *P. parasitica*, 1 cm root segments were taken, stained with FDA, and the fluorescence intensity was detected with a microplate reader (excitation wavelength: 490 nm; emission wavelength: 527 nm). Relative cell death rate represents 100% minus the relative cell viability rate, which was calculated by dividing the fluorescence intensities of roots treated with *P. parasitica* by those treated with mock solution. For each sample, at least 8 plants were analyzed. Three independent experiments were performed. Error bars indicate SD from 8 plants. Asterisks indicate significance analyzed by Student’s t test (*P < 0.05; ***P < 0.001; ****P < 0.0001).

To investigate to what extend is the expression of *BI-1* regulated by *RTP1* during the early colonization of *P. parasitica*, 2-wk-old WT Col-0 and *rtp1* mutant seedlings were either inoculated with *P. parasitica* zoospores or treated with mock solution, and the transcript level of *BI-1* in both WT Col-0 and *rtp1* mutant roots were analyzed at 0, 3, 6, 12 and 24 hpi, respectively, by reverse transcription quantitative PCR (RT-qPCR). We found that the level of *BI-1* transcript in Col-0 was elevated from 3 to 24 hpi, representing its induction during the early infection stage. In comparison, the transcription of *BI-1* in *rtp1* mutants was significantly increased, particularly at 3, 6 and 12 hpi (Fig. 1a). These results indicate that *RTP1* might contribute to negatively affect the expression of *BI-1* during the early colonization of *P. parasitica*.

To examine whether the early induction of *BI-1* is associated with rapid cell death in *P. parasitica*-infected *rtp1* mutants, we performed a fluorescein diacetate (FDA)-based cell viability assay on *P. parasitica*-infected roots of WT Col-0, *rtp1* as well as *rtp1 bi-1* mutant plants at 0, 3, 6 and 12 hpi, respectively. Through analysis on the cell death rate in infected root tissues at indicated time points, as expected, we found significant increase of cell death rate in *rtp1* mutant plants relative to WT Col-0 (Fig. 1b). Notably, less cell death rate was notably detected in *rtp1 bi-1* than *rtp1* mutant plants (Fig. 1b), suggesting a role of *BI-1* in the *RTP1*-mediated cell death in response to attempted infection by *P. parasitica*, particularly at its early biotrophic colonization stage.

### *BI-1* is required for *rtp1*-mediated resistance upon colonization of *P. parasitica*

**Fig. 2:**
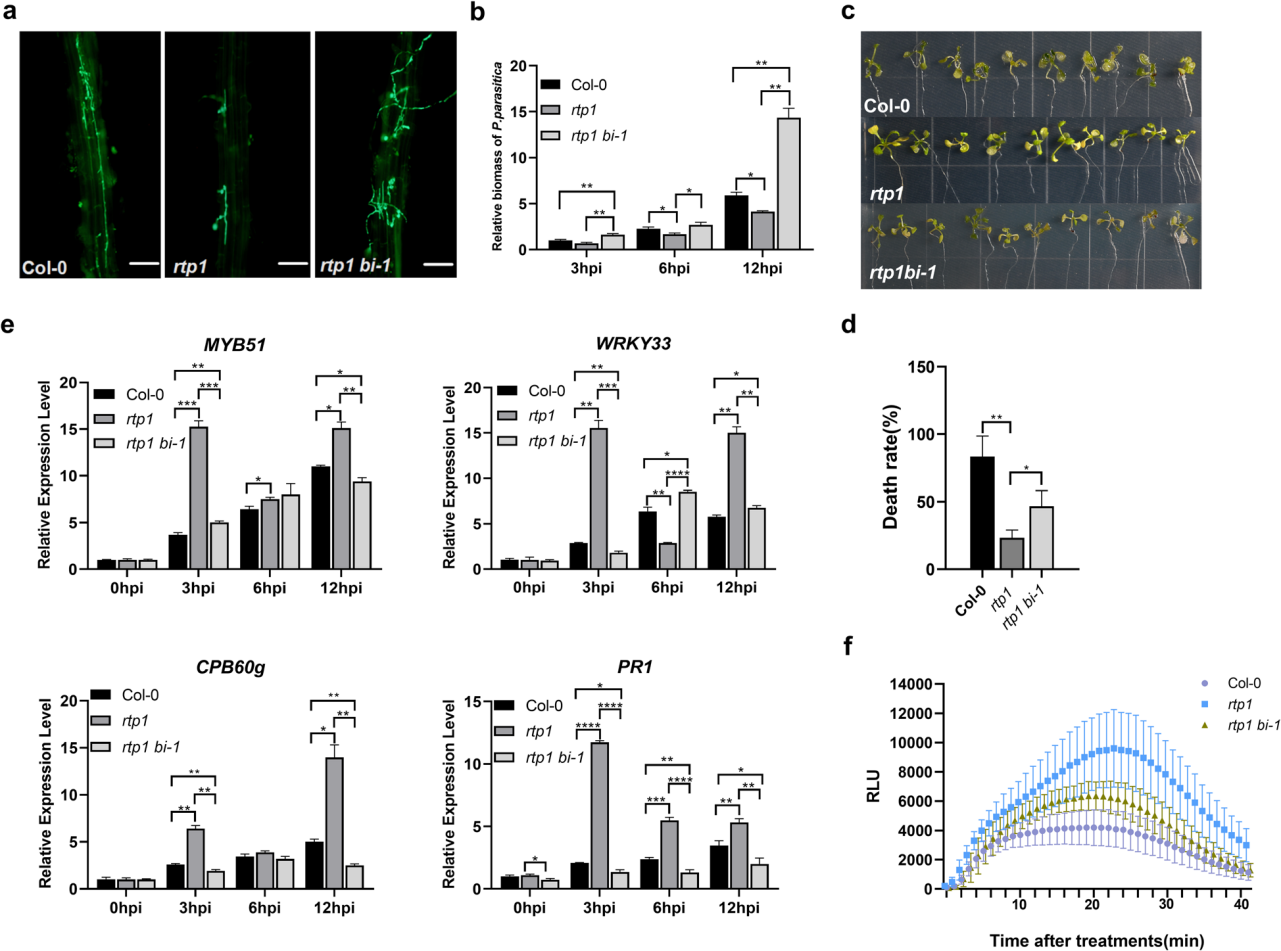
*BI-1* is required for *rtp1*-mediated resistance during *P. parasitica* colonization. **a** The fluorescence microscopic image of roots of 2-wk-old WT Col-0, *rtp1* and *rtp1 bi-1 mutant* infected by GFP-tagged *P. parasitica* transformant, at 12 hpi. Scale bar, 200 μm. At least 5 plants were analyzed. **b ***P. parasitica* biomass in infected roots of WT Col-0, *rtp1* and *rtp1 bi-1* mutant plants at 3, 6 and 12 hpi, was determined by qPCR. *P. parasitica* biomass in all lines was normalized to that in WT Col-0 at 3 hpi (set to 1). Error bars indicate SD of three biological replicates. Asterisks indicate significance analyzed by Student’s t test (*P <0.05; **P < 0.01). **c** Symptoms of root infection by *P. parasitica* zoospores in 2-wk-old WT Col-0, *rtp1* and *rtp1 bi-1* mutant at 7 dpi. **d** The ratios of dead plants in **c** were analyzed. For each sample, at least 10 plants were analyzed. Three independent experiments were performed. Error bars indicate SD from three independent experiments. Asterisks indicate significance analyzed by Student’s t test (*P < 0.05; **P < 0.01). **e** Expressions levels of ER stress-responsive immune genes were determined by qRT–PCR. Roots of 2-wk-old plants of WT Col-0, *rtp1* and *rtp1 bi-1* mutant plants were inoculated with *P. parasitica* zoospores or treated with mock solution. Total RNA was extracted from roots at 0, 3, 6, and 12 hpi. *AtUBC9* was used as the plant reference gene. Three independent experiments were performed. Error bars indicate SD of three biological replicates. Asterisks indicate significance analyzed by Student’s t test (*P < 0.05; **P < 0.01; ***P < 0.001; ****P < 0.0001). **f** ROS burst upon flg22 treatment of leaves of 4-wk-old WT Col-0, *rtp1* and *rtp1 bi-1* mutant plants. At least 12 leaves from 6 plants of each group were measured using a luminol-based chemiluminescence assay. Error bars indicate SD from 12 leaves. RLU, relative light units.

To further elucidate whether *BI-1* is of importance for *rtp1*-mediated resistance against *P. parasitica*, we firstly evaluated the colonization of *P. parasitica* on roots of WT Col-0, *rtp1* and *rtp1 bi-1* mutant plants during the early biotrophic colonization of *P. parasitica*. The microscopy images showed less colonization in *rtp1* than Col-0, while more colonization in *rtp1 bi-1* than *rtp1* (Fig. 2a). Further qPCR results confirmed notably reduced biomass of *P. parasitica* in *rtp1* mutant than in Col-0 at 6 hpi and 12 hpi, respectively (Fig. 2 b). By contrast, significantly more pathogen’s biomass was observed in *rtp1 bi-1* than in *rtp1* plants, from 3 to 12 hpi (Fig. 2 b). Thereafter, we further monitored the root susceptibility in respective plants, at 7 dpi, representing the necrotrophic colonization stage (Fig. 2c). The results showed that over 83% of WT Col-0 seedlings were heavily infected and colonized with *P. parasitica*, whereas more than 60% of *rtp1* plants remained healthy, indicative of increased resistance to *P. parasitica*. In contrast, *rtp1 bi-1* exhibited compromised resistance relative to *rtp1* plants, with significantly elevated seedlings’ death rate (Fig. 2d). Together, these results suggest that *BI-1* plays a crucial role in *rtp1*-mediated resistance against *P. parasitica*.

As previously reported, the expression of several ER stress-responsive immune genes (eg. *WRKY33*, *CBP60g*, *MYB51*) as well as *PR1* was much more strongly induced in *rtp1* mutants than in WT Col-0 upon early infection by *P. parasitica* (Pan et al. 2016; Qiang et al. 2021), we next analyzed the kinetic expression of respective immune genes in roots of WT Col-0, *rtp1* and *rtp1 bi-1* mutant plants during the early biotrophic colonization phase. In comparison to the stronger induction of expression of immune genes *MYB51, WRKY33*, *CBP60g* and *PR1* in *P. parasitica-*colonized *rtp1* plants, their transcript levels were notably reduced in *rtp1 bi-1* double mutants during the early colonization of *P. parasitica* (Fig. 2e). These results support the notion that *BI-1* is at least partially required for the induction of ER stress-responsive immune genes in *rtp1* mutant plants upon early infection by *P. parasitica*.

To further investigate to what extent *BI-1* is involved in the rapid oxidative burst in *RTP1* loss-of-function mutant, we assessed the oxidative burst in leaves of WT Col-0, *rtp1* and *rtp1 bi-1* mutant plants triggered by flg22. The luminol-based assay demonstrated that in comparison to the stronger transient oxidative burst in *rtp1* mutants than in WT Col-0, the *rtp1 bi-1* double mutants showed obviously abolished oxidative burst upon flg22 treatment (Fig. 2f). Taken together, these results suggest that *BI-1* is involved in regulating the expression of ER stress-responsive immune genes and ROS production, which is required for *rtp1*-mediated resistance upon colonization of *P. parasitica*.

### BI-1 positively regulates plant resistance and its accumulation is reduced by RTP1

**Fig. 3:**
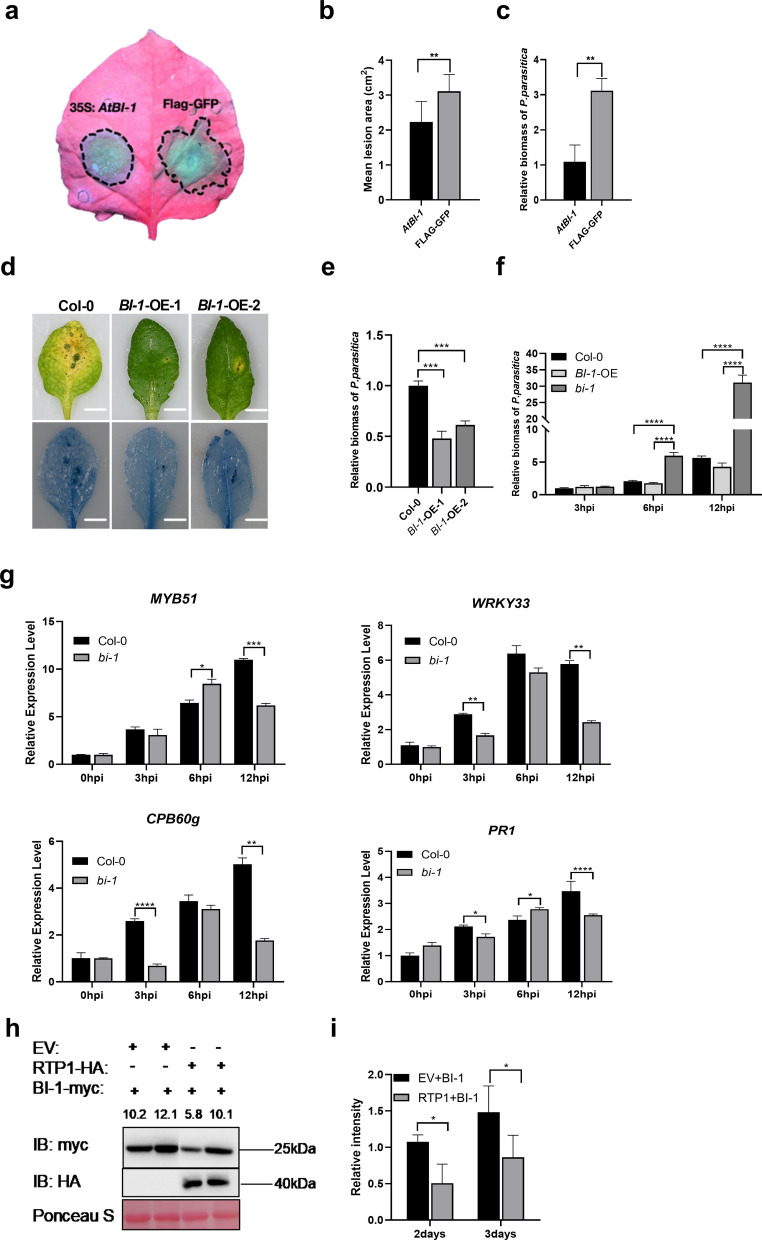
*BI-1* positively affects plant resistance to *P. parasitica* and the protein of BI-1 is destabilized by RTP1. **a** Transient overexpression of 35S::*BI-1* and 35S::Flag-GFP, respectively, in *N. benthamiana* leaves using *Agrobacterium* strains, prior to the inoculation with *P. parasitica* zoospores. Images with ultraviolet irradiation were taken at 48 hpi. **b** The average lesion areas were calculated at 48 hpi. Error bars indicate SD from 10 leaves. Asterisks indicate significance analyzed by Student’s t test (**P <0.01). **c ***P. parasitica* biomass in infected leaves *N. benthamiana* at 48 hpi, as determined by qPCR. Error bars indicate SD from three biological replicates. Asterisks indicate significance analyzed by Student’s t test (**P <0.01). **d** Detached leaves of 4-wk-old WT Col-0 and *BI-1*-OE lines(#1, #2) were inoculated with *P. parasitica* zoospores. The water-soaked lesions with trypan blue staining were photographed at 48 hpi. Scale bar, 0.5 cm. Three independent experiments were performed, and 10 plants per line were analyzed in each experiment. **e ***P. parasitica* biomass in infected leaves of WT Col-0 and *BI-1*-OE lines at 48 hpi, as determined by qPCR. *P. parasitica* biomass in all lines was normalized to that in WT Col-0 (set to 1). Error bars indicate SD from three biological replicates. Asterisks indicate significance analyzed by Student’s t test (***P< 0.001). **f ***P. parasitica* biomass in infected roots of WT Col-0, *BI-1*-OE and *bi-1* mutant at 3 hpi, 6 hpi and 12 hpi, as determined by qPCR. *P. parasitica* biomass in all lines was normalized to that in WT Col-0 at 3 hpi (set to 1). Error bars indicate SD from three biological replicates. Asterisks indicate significance analyzed by Student’s t test (****P< 0.0001) **g** Expressions levels of ER stress-responsive immune genes were determined by qRT–PCR. Roots of 2-wk-old plants of WT Col-0 and *bi-1* mutants were inoculated with *P. parasitica* zoospores or treated with mock solution. Total RNA was extracted from roots at 0, 3, 6, and 12 hpi. *AtUBC9* was used as the plant reference gene. Three independent experiments were performed. Error bars indicate SD of three biological replicates. Asterisks indicate significance analyzed by Student’s t test (*P < 0.05; **P < 0.01; ***P < 0.001; ****P < 0.0001). **h** Protein samples were collected at 2 and 3 days, respectively, post co-infiltration for western blot analysis. The accumulation of BI-1-myc and RTP1-HA was detected with anti-myc and anti-HA, respectively. Ponceau staining of the membrane was used to show equal loading.Two independent experiments were performed and showed similar results. See Supplementary Fig. S1. **i** The relative intensity of BI-1-myc (with the value for BI-1-myc coexpressed with RTP1-HA at 2 dpi set to 1) were determined by ImageJ. Error bars indicate SD from two independent experiments. Asterisks indicate significance analyzed by Student’s t test (*P < 0.05).

To confirm the immune function of *BI-1* in response to colonization of *P. parasitica*, we next infiltrated either construct 35S::*BI-1* or 35S::FLAG-GFP as a control into leaves of *N. benthamiana* prior to inoculation with *P. parasitica*. At 48 hpi, significantly smaller infection lesions and less amount of pathogen biomass were observed in the leaf region infiltrated with 35S::*BI-1* relative to control (Fig. 3a-c). To support this, the detached leaves of 6-week-old *BI-1*-OE transgenic plants were compared with WT Col-0 plants following inoculation with *P. parasitica*. In comparison to the severe water-soaked lesions on leaves of Col-0, at 48 hpi, *BI-1*-OE leaves displayed less visible lesions (Fig. 3d), with significantly reduced *P. parasitica* biomass (Fig. 3e). Consistently, roots of *BI-1*-OE plants exhibited reduced colonization relative to Col-0, during the early infection stage (Fig. 3f). To complement this, more susceptibility in *bi-1* mutants was observed, with significantly increased amount of *P. parasitica* biomass relative to WT Col-0 at 6 hpi and 12 hpi, respectively (Fig. 3f). In accordance with this, compared with the induction of immune genes *MYB51*, *WRKY33*, *CBP60g* and *PR1* in Col-0 upon early colonization of *P. parasitica*, the transcript levels of these genes were notably reduced in infected *bi-1* mutant plants (Fig. 3g). Together, these results imply that *BI-1* might play a positive role in regulating plant resistance and ER stress-associated immunity in response to *P. parasitica* infection*.*

As demonstrated that RTP1 physically interacts with BI-1 (Qiang et al. 2021), we were prompted to examine whether the protein stability of BI-1 is affected by RTP1. For this, the 35S::*BI-1-myc* construct was co-transformed with 35S::*RTP1-HA* or the empty vector (EV) into *N. benthamiana* leaves by agroinfiltration. At 2 and 3 dpi, the leaf proteins were extracted and an equal amount of protein per sample was used to evaluate the impact of RTP1 on BI-1 protein stability. Immunoblotting analysis revealed that BI-1 accumulation was significantly reduced when co-expressed with RTP1 rather than with the EV, with decrease of approximate 57% and 62% accumulation of BI-1 protein at 2 dpi and 3 dpi, respectively (Fig. 3h-i; Supplementary Fig. S1). These results indicate that BI-1 protein accumulation is significantly reduced in the presence of RTP1.

### The promoter of *BI-1* is co-activated by ER stress-associated transcription factors bZIP60 and bZIP28

**Fig. 4:**
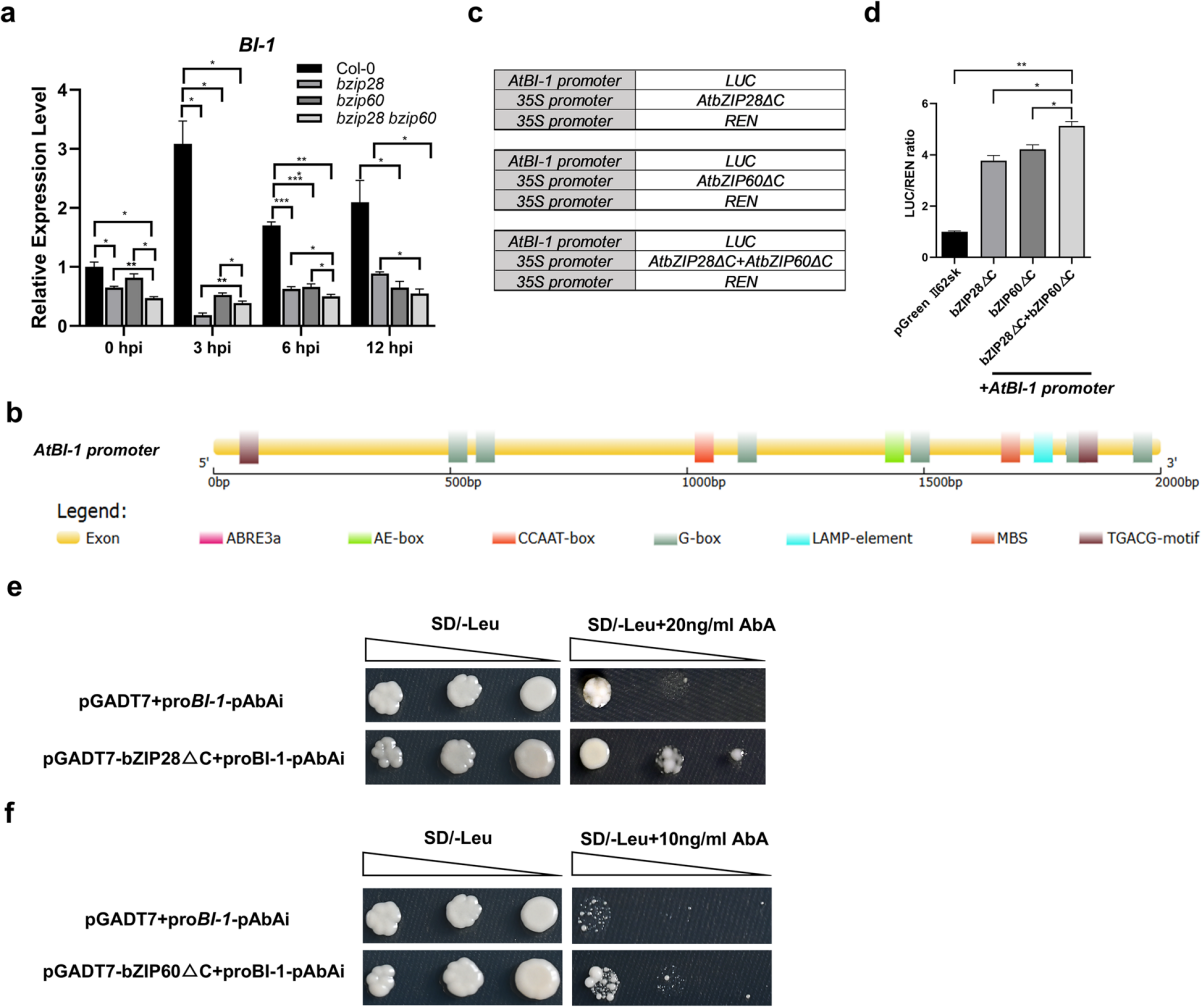
The expression of *AtBI-1* is regulated by ER stress-associated transcription factors bZIP60 and bZIP28. **a** Expressions levels of *BI-1* was determined by qRT–PCR. Roots of 2-wk-old plants of WT Col-0, *bzip28*, *bzip60* and *bzip28bzip60* mutant were inoculated with *P. parasitica* zoospores or treated with mock solution. Total RNA was extracted from roots at 0, 3, 6, and 12 hpi. *AtUBC9* was used as the plant reference gene. Three independent experiments were performed. Error bars indicate SD of three biological replicates. Asterisks indicate significance analyzed by Student’s t test (*P < 0.05; **P < 0.01; ***P < 0.001). **b** Schematic diagram of regulatory elements in the *AtBI-1* promoter. The promoter sequence of the *AtBI-1* gene was analyzed using the promoter prediction PlantCARE Website (http://bioinformatics.psb.ugent.be/webtools/plantcare/html/). **c** A schematic diagram of the vectors structure containing either bZIP28ΔC or bZIP60ΔC and the *AtBI-1* promoter. **d ***AtBI-1* promoter-LUC activity was determined by dual-luciferase reporter assay. A mixture of *A. tumefaciens* containing either bZIP28ΔC, bZIP60ΔC, bZIP28ΔC and bZIP60ΔC or pGreenII62sk and *AtBI-1* promoter were co-infiltrated into leaves of *N. benthamiana*. At 48 h, firefly luciferase (LUC) and renilla (REN) luciferase were assayed. The ratio of LUC/REN to the pGreenII62sk with promoter was used as a calibrator (set as 1). Three independent experiments were performed. Error bars indicate SD of three biological replicates. Asterisks indicate significance analyzed by Student’s t test (*P < 0.05; **P < 0.01). **e-f** Y1H assays on the interaction of the *BI-1 *promoter with bZIP28ΔC (**e**) and bZIP60ΔC (**f**). Oblique triangles above the images denote yeast-cell concentration gradients. The combination of pGADT7 and pro*BI-1*-pAbAi served as a positive control for assay functionality; pGADT7-bZIP28ΔC (**e**) or pGADT7-bZIP60ΔC (**f**) plus pro*BI-1*-pAbAi served as positive controls for the respective interactions. OD_600_ values for dilution series: 0.2, 0.02, 0.002 (**e**); 0.1, 0.01, 0.001 (**f**).

Considering that *BI-1* plays a role in the induction of ER stress-responsive immune genes upon *P. parasitica* infection (Fig. 3e), whose strong induction in *P. parasitica*-infected *rtp1* plants rely on both bZIP60 and bZIP28 (Qiang et al. 2021), we were prompted to explore whether the expression of *BI-1* gene is regulated by the ER stress-associated transcription factors bZIP60 and bZIP28. For this, we first examined the expression of *BI-1* in WT Col-0 and mutants of *bzip28*, *bzip60* as well as *bzip28bzip60,* during the early colonization of *P. parasitica*. The RT-qPCR results showed that *BI-1* could be induced from 3 hpi to 12 hpi in Col-0 (Fig. 4a). By contrast, its induction was notably manipulated in either *bzip28* or *bzip60* mutant plants, and the most significant attenuation of transcript level of *BI-1* was exhibited in *bzip28bzip60* mutant plants (Fig. 4a). These results indicate that the expression of *BI-1* could be co-regulated by bZIP60 and bZIP28, both of which might play a redundant role.

To further explore how bZIP60 and bZIP28 contribute to regulate the expression of *BI-1*, we analyzed the promoter sequence of *BI-1* gene using PlantCare (http://bioinformatics.psb.ugent.be/webtools/plantcare/html/). And the G-box elements required for the binding of bZIP transcription factors, as well as typical ER stress response elements (CCAAT-box) and unfolded protein response core elements (TGACG-motif) were predicted to exist in the promoter region of *BI-1* (Fig. 4b). We therefore examined the effect of either activated form of bZIP28 or bZIP60 TF (i.e. bZIP28ΔC or bZIP60ΔC) on activating the transcription of *BI-1*, using dual-luciferase reporter assay. The promoter of *BI-1* was constructed into pGreenII 0800-LUC as reporter, and the CDS of either bZIP28ΔC or bZIP60ΔC was constructed into pGreenII 62-SK as effector (Fig. 4c). The results demonstrated significant increase of luciferase activities, when either bZIP28ΔC or bZIP60ΔC and the promoter of *BI-1* were co-infiltrated, relative to control (Fig. 4d), indicating that activated form of either bZIP28 or bZIP60 TF could activate the promoter of *BI-1* gene. Intriguingly, in comparison to these, we found notably stronger elevation of luciferase activity when both bZIP28ΔC and bZIP60ΔC were co-infiltrated with *BI-1pro* (Fig. 4d). Furthermore, the yeast one-hybrid assay demonstrated that either bZIP28ΔC or bZIP60ΔC could physically bind to the *BI-1* promoter (Fig. 4e-f). Collectively, these results indicate that both ER stress-associated bZIP60 and bZIP28 TFs could bind to and co-activate the promoter of *BI-1*.

## Discussion

Dominant resistance genes, while effective, are frequently overcome by rapidly evolving pathogen variants, a challenge exemplified by *Phytophthora infestans*. Targeting susceptibility (S) genes offers an alternative route toward durable resistance (Li et al. 2020; Garcia-Ruiz et al. 2021), as illustrated by *GONST1.1* inactivation conferring late blight resistance in tomato without fitness penalty (He et al. 2025). Elucidating the mechanisms by which *S* genes suppress immunity is therefore essential for their precise deployment in crop breeding.

The S gene *RTP1* encodes an ER membrane protein whose loss of function enhances Arabidopsis resistance to *P. parasitica*, accompanied by accelerated cell death, ROS production, and induction of *PR1* and ER stress-responsive immune genes (Pan et al. 2016; Qiang et al. 2021). Yet the key executors of RTP1-mediated cell death have remained unclear. Here, we identify *BI-1* as a critical component: its expression is markedly elevated in *rtp1* mutants (Fig. 1a), and cell death triggered during early infection is significantly attenuated in *rtp1 bi-1* double mutants (Fig. 1b). These data place *BI-1* downstream of *RTP1* in the regulation of cell death. Our previous work established that the vacuolar processing enzyme γVPE is similarly required for *rtp1*-mediated cell death (Gao et al. 2023). Notably, both BI-1 and γVPE have been implicated in ER stress-associated cell death (Watanabe and Lam 2008; Qiang et al. 2012), yet they occupy distinct subcellular compartments, BI-1 at the ER and γVPE in the vacuole. This spatial separation suggests that RTP1 coordinates parallel branches of cell death signaling that converge to regulate immunity.

Beyond its role in cell death, *BI-1* is essential for *rtp1*-mediated resistance. Pathogen colonization was restored in *rtp1 bi-1* double mutants (Fig. 2a-d), and the enhanced expression of ER stress-responsive immune genes and flg22-triggered ROS burst observed in *rtp1* were both abolished in *rtp1 bi-1* double mutant (Fig. 2e, f). Thus, *BI-1* functions as an integral node linking *RTP1* to immune outputs. As VPE is also of importance for *rtp1*-mediated resistance (Gao et al. 2023), both of which function as cell death regulators downstream of *RTP1*, it is plausible that RTP1 acts as a central node that orchestrates multiple ER stress-associated cell death pathways to fine-tune plant immunity. It makes sense to determine how BI-1 and γVPE, spatially distinct regulators, are coordinated by RTP1 to fine-tune immunity in the future work.

The immune function of *BI-1* is conserved across plant-pathogen interactions. Overexpression of *BI-1* enhances resistance to *Magnaporthe oryzae* in rice(Matsumura et al. 2003; Ishikawa et al. 2015), while silencing of *TaBI-1* increases wheat susceptibility to *Puccinia striiformis*(Wang et al. 2012). Moreover, BI-1 cooperates with the IRE1/bZIP60 UPR pathway to restrict viral systemic movement (Gaguancela et al. 2016), reinforcing the link between BI-1 and ER stress signaling. Consistent with these observations, *BI-1* loss-of-function mutants exhibited heightened susceptibility to *P. parasitica* and impaired induction of ER stress-responsive immune genes (Fig. 3a-c, f-g), supporting a positive role for *BI-1* in ER stress-associated immunity.

A physical interaction between RTP1 and BI-1 has been documented (Qiang et al. 2021), and our data demonstrate that RTP1 co-expression markedly reduces BI-1 protein accumulation (Fig. 3h-i). To determine whether this reduction proceeds via the 26S proteasome, we employed the proteasome inhibitor MG132. Proteasome inhibition failed to restore BI-1 levels (Fig. S2), indicating a proteasome-independent destabilization mechanism. Given that BI-1 interacts with ATG6 and both proteins localize to the ER (Xu et al. 2017), RTP1 likely directs BI-1 toward vacuolar or autophagic degradation. Intriguingly, the ER-localized cytochrome P450 CYP71B3 was recently shown to be destabilized by RTP1 in a residue-specific manner (Wei et al. 2025). Whether BI-1 and CYP71B3 form a complex or compete for RTP1 binding remains to be investigated. Elucidating the structural determinants of these interactions may inform molecular design strategies for engineering durable resistance.

We further investigated the transcriptional regulation of *BI-1*. Analysis on *BI-1* promoter revealed bZIP TF binding sites and ER stress-responsive elements (Fig. 4b). Consistent with a role for UPR transducers in *BI-1* expression, induction of *BI-1* upon *P. parasitica* infection was significantly reduced in *bzip60* and *bzip28* single mutants, with the greatest reduction in the *bzip28bzip60* double mutant (Fig. 4a). Dual-luciferase and yeast one-hybrid assays confirmed that the activated forms of bZIP60 and bZIP28 directly bind and co-activate the *BI-1* promoter (Fig. 4c-f). These results establish *BI-1* as a transcriptional target of the canonical ER stress signaling pathway. Notably, *CYP71B3* is also regulated by bZIP60 (Wei et al. 2025), suggesting a convergent regulatory logic wherein RTP1 suppresses immunity by reducing the accumulation of ER-localized, ER stress-induced immune factors.

**Fig. 5:**
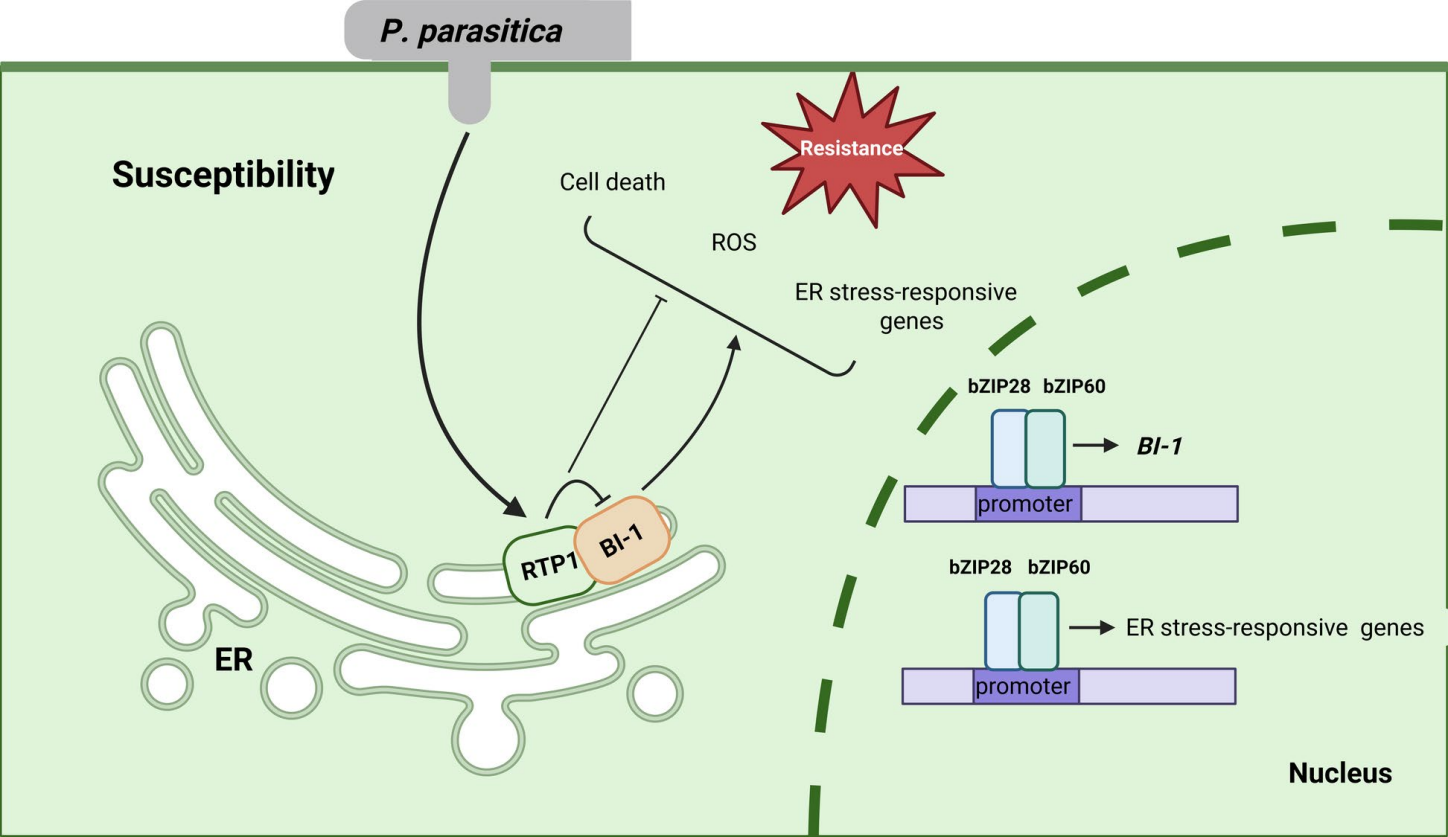
A working model for the regulation of BI-1-mediated resistance by RTP1 and ER stress signaling. The cell death regulator BI-1 confers resistance to *P. parasitica* in Arabidopsis and is antagonized by the susceptibility factor RTP1. *BI-1* promotes *RTP1*-mediated cell death, ROS production, and the expression of ER stress-responsive immune genes. RTP1 physically interacts with BI-1 and reduces its protein accumulation through a proteasome-independent mechanism. Concurrently, the activated forms of the ER stress transducers bZIP60 and bZIP28 bind and co-activate the *BI-1* promoter, thereby potentiating *BI-1* transcription and downstream immune outputs. This dual-level regulation, transcriptional activation by the UPR and post-translational destabilization by RTP1, positions BI-1 at a key nexus of ER stress-associated immunity.

Collectively, our findings reveal that BI-1 is essential for RTP1-mediated cell death and immunity and operates at two regulatory levels: it is transcriptionally activated by the ER stress transducers bZIP60 and bZIP28, and post-translationally antagonized by RTP1 through proteasome-independent destabilization (Fig. 5). This dual control positions BI-1 at the intersection of ER stress sensing and immune execution. The identification of additional RTP1 targets of γVPE and CYP71B3 implies that RTP1 functions as a central hub that coordinates multiple branches of ER-associated immunity. Future dissection of the interplay among these factors will deepen our understanding of how *S* genes suppress defense and will inform the rational design of broad-spectrum disease resistance in crops

## Conclusion

Our study reveals a multifaceted regulatory network wherein the susceptibility factor RTP1, the cell death modulator BI-1, and the ER stress transducers bZIP60 and bZIP28 converge to govern plant immunity. We identify BI-1 as a key executor of Arabidopsis resistance to *Phytophthora parasitica* that functions through an ER stress-associated immune pathway. RTP1 physically interacts with and destabilizes BI-1, representing a post-translational mechanism by which this *S* gene suppresses defense. Concurrently, bZIP60 and bZIP28 activate *BI-1* transcription, placing this cell death regulator under direct control of the canonical UPR. The shared transcriptional regulation of *BI-1* and *CYP71B3* further reinforces the central role of ER stress signaling in RTP1-governed immunity. Together, these findings refine our understanding of how ER stress interfaces with cell death and defense, and they highlight promising targets for engineering durable disease resistance in crops. Future work should delineate the precise hierarchy of bZIP60/bZIP28-orchestrated signaling and elucidate how ER stress-responsive cell death factors coordinately shape broad-spectrum pathogen resistance.

## Materials and methods

### Plant materials, growth conditions, and plant inoculation

The Arabidopsis* RTP1* T-DNA insertion line (SALK_094320) was obtained from the Arabidopsis Biological Resource Center (ABRC). The *bzip28* and *bzip60* mutants were kindly provided by Dr. Patrick Schäfer (University of Giessen, Germany); the *bi-1* mutant and *BI-1*-OE (#1, #2) transgenic plants were kindly provided by Dr. Ruth Eichmann (University of Giessen, Germany). The double mutant of *bzip28bzip60* was provided by Dr Han Jiajia (Yunnan University, China). The double mutant of *rtp1 bi-1* was generated by crossing *rtp1* with *bi-1*. The T-DNA insertion homozygous mutants were confirmed by PCR using primers rtp1-LP, rtp1-RP, bi-1-LP, bi-1-RP and LBb1.3 (Supplementary Fig. S3 and Table S1). For root inoculation, all Arabidopsis seeds were sterilized and grown in squared dishes on half-strength MS. Roots of 2-wk-old seedlings were dip-inoculated with *P. parasitica* zoospores (Pan et al. 2016). For detached leaf inoculation on leaves of *A. thaliana* and *N. benthamiana*, the plants were grown at 23℃ with 16 h of light per 24 h. The detached leaf inoculation was conducted as previously described (Qiang et al. 2021). The culture and zoospore production of *P. parasitica* were conducted as previously described (Wang et al. 2011). The concentration of zoospores was adjusted to 10^5^ zoospores/mL. For the quantitation of infection, genomic DNA was extracted by the CTAB method. The method to quantify the pathogen’s biomass was conducted as previously described (Qiang et al. 2021).

### Plasmid construction

*BI-1* promoter from genomic DNA was inserted into pGreenII 0800-LUC with BamHI and HindIII sites, and bZIP28ΔC, bZIP60ΔC and both of bZIP28ΔC and bZIP60ΔC were inserted into pGreenII 62SK vector with BamHI and PstI sites. To create myc-fusion plasmids, the myc fragment was cloned into pKannibaI (Wesley et al. 2001) with Xho I and EcoR I sites and Not I sites were used to release the fragment with the promoter and terminator and then inserted into the binary vector pART27 (Gleave 1992). The mature BI-1 coding sequences was inserted into previously modified pART27 at the EcoR I and Xba I sites to create BI-1-myc. The fusion fragments RTP1-HA were obtained through overlapping PCR and inserted into the monoclonaI site of pKannibaI (RTP1-HA: EcoR1 and XbaI), and thereafter inserted into the binary vector pART27 at the NotI site. For other plant expression constructs, including 35S:*AtBI-1* and FLAG-GFP, fusion fragments were obtained from overlapping PCR and cloned into the EcoR I and Xba I sites of the binary vector pART27 under the 35S-promoter constructs.

### Gene expression analysis

Total RNA was extracted from root material by using TRIzol (Invitrogen) reagent. For RT-qPCR analysis, cDNA was synthesized from 1µg of total RNA using PrimeScript RT reagent Kit (TaKaRa). Twenty nanograms of cDNA were used as template for the amplification of candidate genes using SYBR premix Kit (Roche) according to the manufacturers’ instructions. Primers used were listed in Supplemental Table S1. The Ct values of genes were quantified using an iQ7 Real-Time Cycler (Life technologies, USA). Expression fold changes were calculated by the 2^ΔΔ^Ct method (Schmittgen and Livak 2008).

### *Agrobacterium*-mediated transient expression in leaves of *N. benthamiana*

*Agrobacterium tumefaciens* GV3101 strain harboring the recombinant constructs were grown for 16 h at 28 °C in LB medium in the presence of corresponding antibiotics. Thereafter, the bacterial cells were harvested by centrifugation, and resuspended in MES buffer to OD_600_ =0.4 and incubated for 1 h before infiltration. The 4-wk-old *N. benthamiana* leaves were infiltrated using 1 mL syringe without needle from the abaxial side. And 2 d after infiltration, leaves were collected to inoculate with *P. parasitica*. Lesion area was evaluated at 48 h post infiltration (hpi).

### Dual-luciferase reporter assay

Respective transcription factors and promoter of *BI-1* were transiently overexpressed in *N. benthamiana*. After two days of co-expression, 0.1 g of leaf samples were collected, carefully avoiding the leaf veins. These samples were placed into 2 mL centrifuge tubes containing 4 steel beads. The samples were immediately snap-frozen in liquid nitrogen. Subsequently, they were homogenized into a fine powder by shaking. Then, 500 μL of Passive Lysis Buffer was added to the tubes, followed by vortex-shaking. The tubes were centrifuged at 12,000 rpm for 5 min at 4°C. The supernatant was then transferred to a new centrifuge tube. Twenty microliters of the supernatant was added to each well of a white microplate reader plate. Three replicates were set for each sample. Next, 50 μL of firefly luciferase reaction working solution was added to each well. Chemiluminescence was detected using a microplate reader. After the measurement, 50 μL of Renilla luciferase reaction solution was further added to each well of the microplate. Chemiluminescence was then detected using the Varioskan LUX multimode reader, and the relative fluorescence intensity was calculated.

### Yeast one-hybrid (Y1H)

Y1H assays were performed using the Matchmaker Gold Y1H System (Clontech, USA) according to the manufacturer's instructions. The Coding DNA Sequence (CDS) of AtbZIP28ΔC and AtbZIP60ΔC were cloned and inserted into pGADT7 to construct the prey vectors, and the promoter sequence of *AtBI-1* was cloned and inserted into the pAbAi vector to construct the bait vector. The bait construct was linearized and transformed into the Y1H Gold yeast strain to generate the bait reporter strain. The prey plasmids were subsequently introduced into the respective bait strains. Transformants were selected on synthetic dropout medium SD/-Leu. For the interaction assay, yeast clones were cultured and spotted onto SD/-Leu medium supplemented with or without aureobasidin A (AbA) at the indicated concentrations (20 ng/mL for bZIP28ΔC; 10 ng/mL for bZIP60ΔC). For the dilution series shown in the figures, yeast cells were adjusted to the following concentration gradients (OD_600_) before spotting: 0.2, 0.02, and 0.002 for bZIP28ΔC; and 0.1, 0.01, and 0.001 for bZIP60ΔC.

### Determination of cell death rate

Cell death was assessed at two distinct stages of *P. parasitica* infection. For early infection stage analysis (0–12 hpi), roots of 2-wk-old WT Col-0, *rtp1*, and *rtp1 bi-1* mutants infected with *P. parasitica* were stained with FDA. Roots were washed with PBS buffer before transferred to the PBS buffer containing FDA at a final concentration of 5 μmol L^–1^. After incubation for 15 min, roots were washed twice by the same buffer, fluorescence intensities were measured at 527 nm after excitation at 490 nm by the Varioskan LUX multimode reader.

For necrotrophic infection stage analysis (7 dpi), disease symptoms and plant death were evaluated. Roots of 2-wk-old seedlings of WT Col-0, *rtp1*, and *rtp1 bi-1* mutants grown on square dishes containing half-strength MS medium were dip-inoculated with *P. parasitica* zoospores. Disease symptoms were monitored and photographed at 7 dpi. The ratio of dead plants was calculated as the number of dead plants divided by the total number of inoculated plants per genotype. For each genotype, at least 10 plants were analyzed per experiment.

### Fluorescence microscopy assay

For fluorescence microscopy analysis, roots of 2-wk-old plants of WT Col-0, *rtp1*, and *rtp1 bi-1* mutants grown on square dishes containing half-strength MS medium were dip-inoculated with zoospores prepared from the GFP-tagged *P. parasitica* transformant. At 12 hpi, the infected roots were collected and mounted on glass slides with sterile water. Fluorescence images were captured using a fluorescence microscope equipped with a GFP filter. For each sample, at least five plants were analyzed.

### ROS burst assay

Approximately 20 leaves from different Arabidopsis plants were cut and punched with a 6 mm diameter puncher. The leaf disks were placed on a small amount of sterile water with the abaxial side up at room temperature and floated overnight to balance the reactive oxygen species burst caused by punching. The next day, 100 μL of luminol reagent and 100 μL of 500-fold diluted HRP stock were mixed to form the assay buffer. The 200 μL of assay buffer was added to each well of a white 96-well plate, and the leaf discs were gently placed in, avoiding any damage. The multimode reader was turned on, and the relevant parameters were set. Thereafter, 5 μL of 41 μmol/L flg22 was added to each well of the microplate, and the luminescence signal kinetics of the leaves were immediately detected. Afterwards, 30–50 cycles were set with an interval of 1 min, and 12 leaves from 6 plants of each group were measured.

### Co-IP and immunoblot assays

*N. benthamiana* leaves were harvested at 2 d after agroinfiltration, and proteins were extracted with HEPES buffer. For proteasome inhibition assays, leaves were infiltrated with 50 μM MG132 at 12 h prior to sample collection to block proteasome-mediated degradation. Protein stability of BI-1-myc co-expressed with RTP1-HA or empty vector (EV) was analyzed by immunoblotting (IB). Co-immunoprecipitation (Co-IP) was performed as described (Win et al. 2011; Qiang et al. 2021). After protein samples were boiled for 5 min, the supernatant was separated on SDS–PAGE gels and transferred to polyvinylidene fluoride membranes (Roche). Antibodies used for this study included anti-HA (AE008, ABclonal; 1:5000) and anti-myc (AE010, ABclonal; 1:5000). Ponceau staining served as a loading control. Protein bands were quantified using ImageJ software (Schneider et al. 2012).

### Statistical analysis

Results are expressed as means ± standard deviation (SD) or ± standard error (SE) as indicated in the figure legends and represent at least three biological repetitions. Statistical analysis was performed using Student’s t-test. *P*<0.05 was considered significant.

## Supplementary Information

Supplementary Material 1.

## Data Availability

All data generated or analyzed during this study are included in the published article and its supplementary information files.

## Abbreviations

ER: endoplasmic reticulum
RTP1: Resistance to *Phytophthora parasitica* 1
BI-1: Bax Inhibitor-1
*P. parasitica*: *Phytophthora parasitica*
*P. infestans*: *Phytophthora infestans*
HR: hypersensitive response
PCD: programmed cell death
PICD: plant immunity with cell death
UPR: unfolded protein response
ER-CD: ER stress-induced cell death
IRE1: inositol-requiring enzyme 1
bZIP: basic leucine zipper
*S *genes: susceptibility genes
