# Supplementary material for: Bax inhibitor-1 confers resistance to Phytophthora parasitica and is antagonized by RTP1 in Arabidopsis
Source: Stress Biol. 2026 Jun 23;6(1):46. doi: 10.1007/s44154-026-00318-0 (PMC13291311; doi:10.1007/s44154-026-00318-0)
Supplement: Supplementary file 1 — Supplementary Material 1. [file 44154_2026_318_MOESM1_ESM.docx]

**Title:** Bax inhibitor-1 confers resistance to *Phytophthora parasitica* and is antagonized by RTP1 in *Arabidopsis*

**Journal name**

*Stress biology*

**Authorship**

Yujing Fang^1†^, Jing Zhang^1†^, Xianxian Gao^1^, Shuhan Guo^1^, Xiaoyin Xu^1^, Bianbian Wang^1^, Qing Zheng^1^, Weixing Shan^1*^, Xiaoyu Qiang^1*^

**Affliation and email address of the corresponding author**

^1^State Key Laboratory for Crop Stress Resistance and High-Efficiency Production and College of Agronomy, Northwest A&F University, Yangling, Shaanxi 712100, China

†These authors contributed equally to this work.

*Corresponding authors: Weixing Shan and Xiaoyu Qiang

College of Agronomy and State Key Laboratory for Crop Stress Resistance and High-Efficiency Production, Northwest A&F University, Yangling, Shaanxi 712100, China.

Telephone: +86 29 87080102

E-mail: Weixing Shan, wxshan@nwafu.edu.cn; Xiaoyu Qiang, qiangxiaoyu@nwafu.edu.cn

ORCID: Weixing Shan, https://orcid.org/0000-0001-7286-4041; Xiaoyu Qiang, https:// orcid.org/0000-0001-7819-7737

## Supplementary Figures


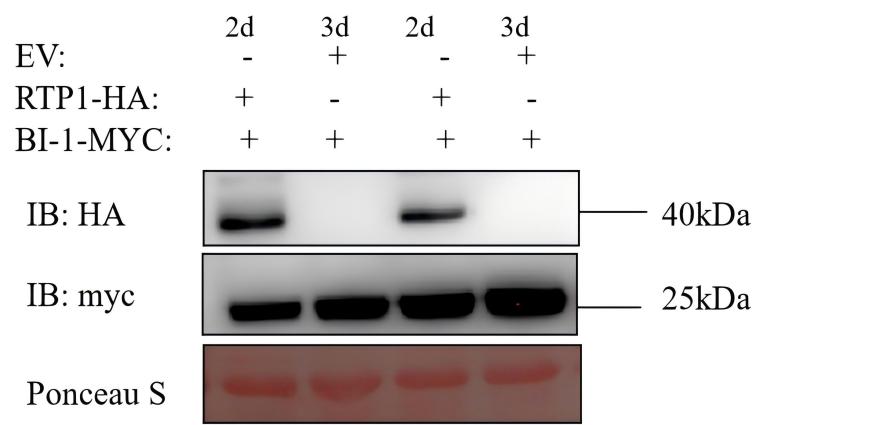


## Supplementary Material 1. Fig. S1. Protein stability of BI-1, co-expressed with RTP1 or EV, was analyzed by immunoblotting (IB). Protein samples were collected at 2 and 3 days , respectively, post co-infiltration, for western blot analysis. The accumulation of BI-1-myc and RTP1-HA was detected with antibody of -myc and -HA, respectively. Protein size markers were indicated in kDa, and protein loading was indicated by Ponceau staining.

##

## Supplementary Material 1. Fig. S2. Protein stability of BI‑1, co‑expressed with RTP1 or EV, was analyzed by immunoblotting (IB). At 12 h prior to sample collection, 50 μM MG132 was infiltrated to inhibit proteasome‑mediated degradation. a Protein samples were harvested at 2 and 3 days post co‑infiltration, respectively, for western blot analysis. Accumulation of BI‑1‑myc and RTP1‑HA was detected with anti‑myc and anti‑HA antibodies, respectively. Protein size markers are indicated in kDa, and Ponceau staining served as a loading control. b The relative intensity of BI-1-myc (protein accumulation at 2-d post infiltration, when coexpressed BI-1-myc and RTP1-HA, was set to 1) were determined by Image J. Data presented show means of two independent experiments ± SD. Asterisks indicate significance analyzed by Student’s t test (***P* < 0.01).

.

##
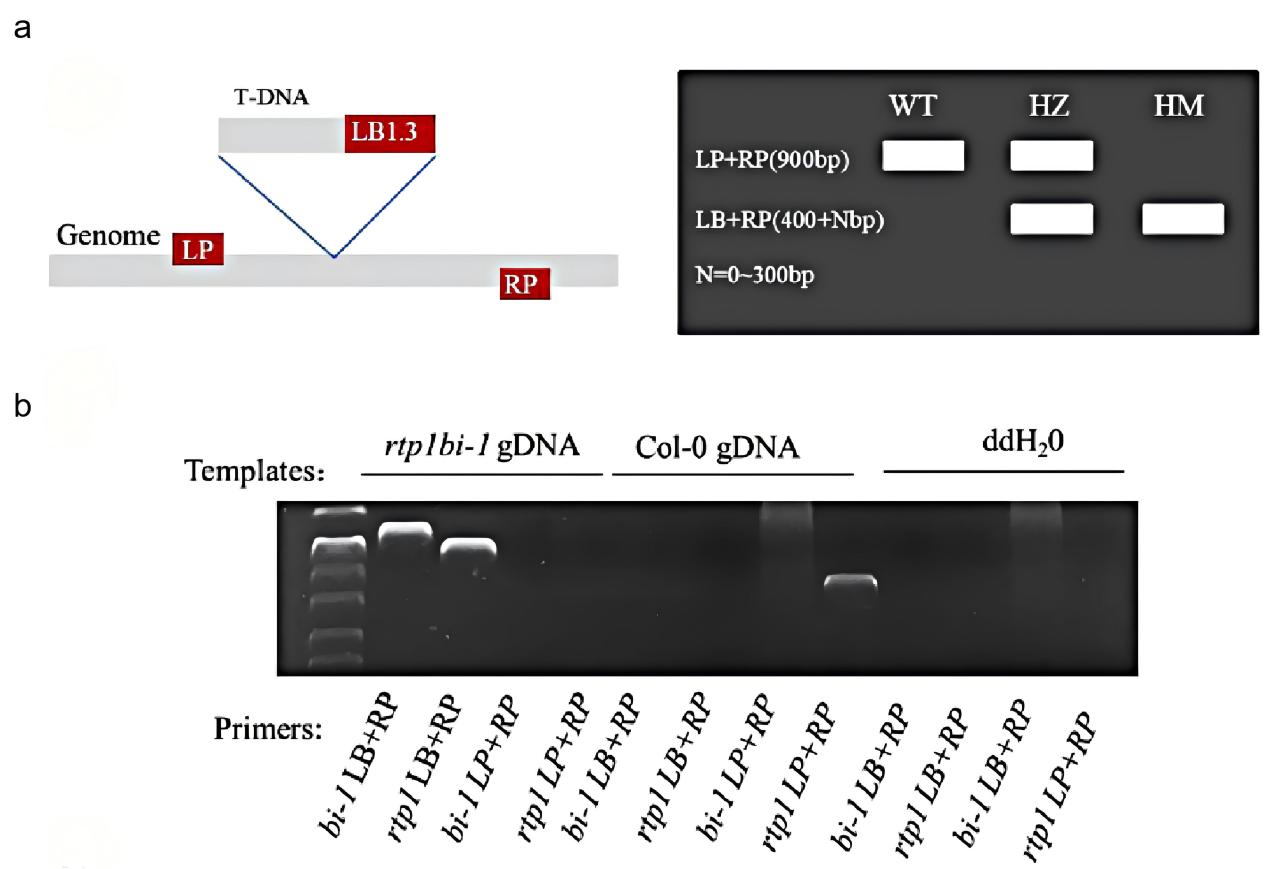


## Supplementary Material 2. Fig. S3. Identification of homozygous plants of the *rtp1bi-1* double mutant. a Schematic of SALK T-DNA primers design. b The homozygote of *rtp1bi-1* double mutant was detected by PCR.

## Supplementary Table 1

Primers used in this study

| **Primer name** | **Primer Sequence (5' - 3')** |
| --- | --- |
| 35S::StBI-1-F | GGAGAGGACACGCTCGAGATGGAAGGTTICACATCGTTCTT |
| 35S::StBI-1-R | TAATTAACCCCATAAGCTTCTAGTTTCTCCTCTTCTTCTCT |
| pGreenII0800-LUC-BI-1-F | GTCGACGGTATCGATAAGCTTTTTTTTGTTAAGATCTCTCTCTCTC |
| pGreenII0800-LUC-BI-1-R | CGCTCTAGAACTAGTGGATCC TGTTTCGTTTTTTTTGCTTGACTTT |
| pGreenII62-SK-bZIP28△C-F | CGCTCTAGAACTAGTGGATCC ATGACGGAATCAACATCCGTG |
| pGreenII62-SK-bZIP28△C-R | CTTGATATCGAATTCCTGCAGTCAAACCTTCTTGAGCTTACTTTTA |
| pGreenII62-SK-bZIP60△C-F | CGCTCTAGAACTAGTGGATCCATGGCGGAGGAATTTGGAAG |
| pGreenII62-SK-bZIP60△C-R | CTTGATATCGAATTCCTGCAGTCAAGACTCCTGCTTCGAC |
| pAbAi-proBI-1-F | TTCGAGCTCGGTACCTACTATTTGATGATGTATTAACAGTAGAAAGCATATTAAAAAAAATAAAA |
| pAbAi-proBI-1-R | TGCCTCGAGGTCGACTTACTGCTTATGTGTAGTTATTACGCATTC |
| pGADT7-bzip28-F | GAGGCCAGTGAATTCATGACGGAATCAACATCCGTG |
| pGADT7-bzip28-R | GAGCTCGATGGATCCTCAGGTGGCTACGAGATGGA |
| pGADT7-bzip60-F | GAGGCCAGTGAATTCATGGCGGAGGAATTTGGAAGC |
| pGADT7-bzip60-R | GAGCTCGATGGATCCTCACGCCGCAAGGG |
| RTP1:HA-F | CGGAATTCATGGAGGTGAAGGTTAGAAGAG |
| RTP1:HA-F | GCTCTAGATTAAGCATAATCAGGAACATCATAAGGATATACAGGACTTTCTTCCTGGT |
| BI-1:myc-F | GAACAGAAACTGATCTCTGAAGAAGATCTGGATGCGTTCTCTTCCTTC |
| BI-1:myc-F | CAGATCTTCTTCAGAGATCAGTTTCTGTTCGTTCTCCTTTTCTTCTTCTTCTC |
| LBb1.3 | ATITTGCCGATITCGGAAC |
| rtp1- LP | CGAACACAAAAGGACTCATCC |
| rtp1- RP | TGAGTTTTGTAGCTGGCAAGG |
| bi -1-LP | CAGAAGCTGGAGCTATGATTC |
| bi -1-RP | TGGGTCAACATCAATTGCCAC |
| qPCRAtUBC9-F | CATCGGATAGCCCTTATTCTG |
| qPCRAtUBC9-R | TGGAACACCTTCGTCCTAAAA |
| qPCRBI-1-F | GCAGCAGCAATGTTAGCAAG |
| qPCRBI-1-R | CACCACCATGTATCCCACAA |
| qPCRMYB51-F | CATCTCTCTTCACGCCCTTC |
| qPCRMYB51-R | TGCCCTTGTGTGTAACTGGA |
| qPCRWRKY33-F | GAAACAAATGGTGGGAATGG |
| qPCRWRKY33-R | TGICGTGIGATGCTCTCTCC |
| qPCRCPB60g-F | GGAAGAAAGCTGGACCGTTG |
| qPCRCPB60g-R | AGTCCAGCTCGAGTTATCCG |
| qPCRPR1-F | CGTTCACATAATTCCCACGAG |
| qPCRPR1-R | TCAGTGAGACTCGGATGTGC |
| AtUBC9-F | TTCATTGGCAGGCCACTAT |
| AtUBC9-R | CTTAGGAGGCTTAAATGGGTAA |
| PPUBC9-F | CCACTTAGAGCACGCTAGGA |
| PPUBC9-R | TACCGACTGTCCTTCGTTCA |
